# Fatal HLH in patients with X-linked lymphoproliferative disease 1 due to a novel variant in *SH2D1A*: case report

**DOI:** 10.3389/fimmu.2025.1602107

**Published:** 2025-05-19

**Authors:** Oksana Boyarchuk, Alla Volokha, Nataliia Yarema, Olga Dyvoniak, Tetyana Tomashivska, Ivanna Shymanska, Halyna Makukh, Jolan E. Walter

**Affiliations:** ^1^ Department of Children's Diseases and Pediatric Surgery, I.Horbachevsky Ternopil National Medical University, Ternopil, Ukraine; ^2^ Department of Pediatrics N1, Shupyk National Healthcare University of Ukraine, Kyiv, Ukraine; ^3^ Department of Pediatric Infectious Diseases, Ternopil City Hospital 2, Ternopil, Ukraine; ^4^ Department of the Research and Biotechnology, Molecular-Genetic Laboratory, Scientific Medical Genetic Center LeoGENE, Lviv, Ukraine; ^5^ Division of Pediatric Allergy and Immunology, Department of Pediatrics, University of South Florida, St. Petersburg, FL, United States; ^6^ Division of Pediatric Allergy and Immunology, Research Center for Primary Immunodeficiencies, Johns Hopkins All Children’s Hospital, St. Petersburg, FL, United States

**Keywords:** XLP1, hemophagocytic lymphohistiocytosis, SH2D1A gene, Epstein-Barr virus, SARS-CoV-2, HLH

## Abstract

**Introduction:**

X-linked lymphoproliferative disease type 1 (XLP1) is an inborn error of immunity (IEI) caused by pathogenic variants in the *SH2D1A* gene, leading to severe immune dysregulation, often triggered by Epstein-Barr virus (EBV) infection. Hemophagocytic lymphohistiocytosis (HLH) is one of the most severe manifestations of XLP1 with high mortality.

**Objective:**

Present a clinical case of fatal HLH associated with a novel *SH2D1A* variant, highlighting the variability of clinical presentation and the potential role of co-infections.

**Methods:**

We analyzed clinical and laboratory data of three brothers who died from HLH in early age. Genetic evaluation was performed using a 576-gene panel for IEI (Veritas, Spain, supported by the Jeffrey Modell Foundation). Alive siblings and parents were tested in Scientific Medical Genetic Center LeoGENE, Ukraine.

**Results:**

A 1-year-old boy was admitted with a persistent 4-day fever and clinical signs of hepatosplenomegaly, anemia, neutropenia, hypertransaminasemia, and hypoproteinemia. Immunophenotyping revealed decreased CD4, increased CD8 T cells, reduced NK cell counts, and elevated immunoglobulin levels. This patient demonstrated high EBV viremia and positive serological markers for SARS-CoV-2. Despite intensive treatment, HLH progressed rapidly, leading to fatality within 35 days. Genetic testing identified a novel, likely pathogenic hemizygous *SH2D1A* variant, c.175delC (p.Thr59Glnfs*22), not previously reported in affected individuals or the gnomAD database. Family history shows that two older male siblings died at 11 months and 1 year 9 months from a rapidly developed disease presented by fever, hepatosplenomegaly, dermatitis, enterocolitis, anemia, thrombocytopenia, and hypertransaminasemia. The second affected sibling tested positive for EBV serology. The family also included a healthy sister and brother, both with positive EBV serology (IgG) but no detectable viremia. Carrier testing confirmed that the mother and sister are heterozygous carriers, while two male siblings (one of them was born 1 month ago) are unaffected.

**Conclusion:**

We identified a novel *SH2D1A* variant associated with fatal HLH in XLP1. Our findings highlight the importance of early genetic diagnosis before EBV exposure to improve patient outcomes. The potential role of co-infections, including SARS-CoV-2, in triggering HLH in XLP1 remains an area for further investigation.

## Introduction

X-linked lymphoproliferative disease type 1 (XLP1), caused by variants in the *SH2D1A* gene, is a rare inborn error of immunity (IEI) characterized by severe immune dysregulation (1). XLP1 occurs in approximately 1 to 2 males per million (2).

The disease was first described by Purtilo et al. in 1974, when they reported six boys from the Duncan family who died between the ages of 2 and 19 years due to lymphoproliferative disease, with three cases presenting infectious mononucleosis as a terminal condition (3). In 1998, Coffey et al. utilized a patient’s deletion in *SH2D1A* as a reference for genetic mapping to identify the XLP locus. Their research confirmed that mutations in *SH2D1A*, rather than EBV infection itself, play a critical role in XLP1 development. This discovery was pivotal in understanding the molecular mechanisms and pathogenesis of the disease (4).

The *SH2D1A* gene is located on the X chromosome and encodes the SLAM-associated protein (SAP) (1, 4). SAP is primarily expressed in T cells and natural killer (NK) cells, where it plays a crucial role in regulating signal transduction pathways downstream of SLAM family surface receptors. This regulation is essential for immune response modulation, particularly in the activation and interaction of lymphocytes. XLP1 results from loss-of-function mutations in *SH2D1A*, leading to impaired regulation and function of CD4+ T cells (and consequently B cells), CD8+ T cells, and NK cells, as well as the development of NKT cells (2).

Notably, NK cells from XLP1 patients are unable to kill Epstein-Barr virus (EBV)-infected B cell lines. This impairment results from inhibitory signals arising from the interaction between 2B4 and CD48. Studies have demonstrated that disrupting the 2B4-CD48 interaction with specific antibodies can restore the lysis of EBV(+) target cells that lack human leukocyte antigen (HLA) class I molecules (5).

The most common clinical manifestations of XLP1 include severe infectious mononucleosis, hemophagocytic lymphohistiocytosis (HLH), lymphoma, and dysgammaglobulinemia. EBV-triggered HLH is one of the most frequent and severe manifestations, often leading to fatal outcomes (6, 7). Immune dysregulation results in excessive cytokine production, a hyperinflammatory response, widespread tissue damage, and multi-organ failure (8, 9). Interestingly, XLP1 phenotypic features may be observed even in patients without serological or molecular evidence of prior EBV infection (10).

Advances in molecular genetics have provided powerful tools for the early detection of genetic abnormalities, allowing for personalized treatment approaches and significantly improving the prognosis for patients with HLH.

The aim of our study was to present a clinical case of fatal HLH associated with a novel *SH2D1A* variant, highlighting the variability of clinical presentation and the potential role of co-infections.

This case is unique due to the identification of a novel, *SH2D1A* variant (c.175delC, p.Thr59Glnfs*22) associated with fatal HLH in XLP1. To our knowledge, this frameshift mutation has not been previously reported in affected individuals or the gnomAD database. The index patient had high EBV viremia along with serological markers for SARS-CoV-2, suggesting a multifactorial disease trigger. These findings align with prior research on the role of EBV in XLP1-associated HLH but expand current knowledge by demonstrating a possible impact of additional viral infections in exacerbating immune dysregulation (11).

## Materials and methods

### Clinical evaluation

Clinical evaluation, blood cell, and immunological examinations were conducted. The study adhered to the principles of the 1975 Declaration of Helsinki (as amended in 2000) and received approval from the Ethics Committee of I. Horbachevsky Ternopil National Medical University. Informed consent was obtained from the legal guardians of all participants.

### Blood cells and immunological studies

Routine hematological assays were performed for complete blood cell analysis. Peripheral blood mononuclear cell lymphocyte subsets were identified via flow cytometry. Monoclonal antibodies were used to detect cell surface markers, including CD3, CD4, CD8, CD19, CD16, and CD56. Serum levels of IgG, IgA, IgM were measured using standard immunological techniques.

### Whole exome sequencing and panel sequencing

A primary immunodeficiency panel that included 576 genes based on whole-exome sequencing (Veritas, Spain) was performed. Siblings and parents were tested in Scientific Medical Genetic Center LeoGENE, Lviv, Ukraine, by the Sanger sequencing method. This variant is not described in databases, but according to ACMG criteria PVS1, PM4, PP4, it can be classified as pathogenic (1 very strong (PVS1, 1 Moderate (PM4), and 1 supporting (PP4)) (12).

## Results

### Case presentation

A 2-year-old boy (affected sibling 3) was hospitalized at the city hospital with complaints of fever up to 39°C, pallor of the skin and mucous membranes, drowsiness, and loss of appetite. The illness began 4 days before admission with a fever.

The child was from the fifth pregnancy, delivered at 38 weeks of gestation through the fifth physiological delivery. The parents are not consanguineous. The patient was of Caucasian ethnicity and Ukrainian origin. He was born with a weight of 3500g. He had two respiratory viral infections that presented as bronchitis and was hospitalized once for this.

**Figure 1:**
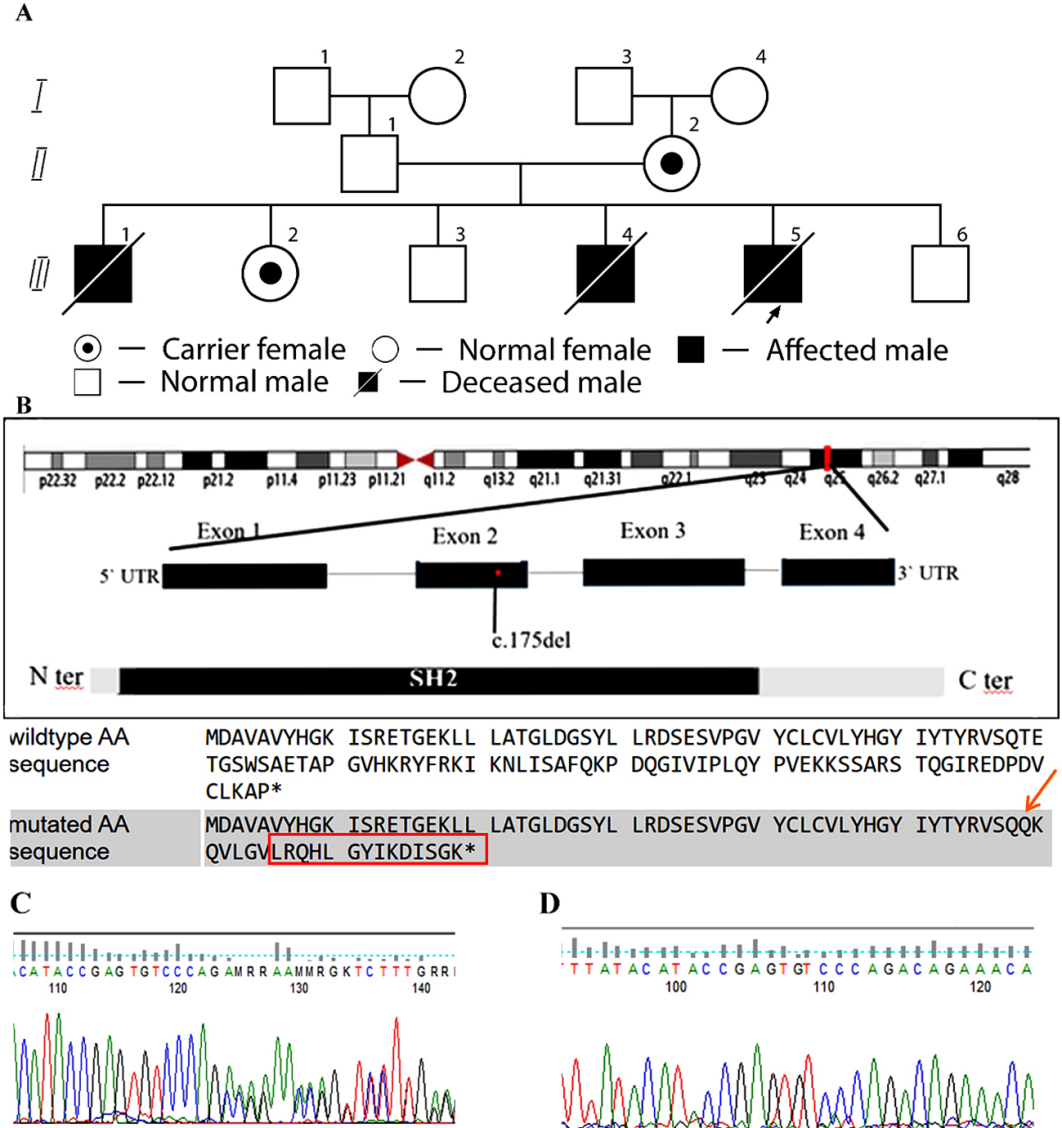
Family pedigree and structure of the *SH2D1A* gene. **(A)** Pedigree of a family with XLP1. Arrows indicate index case. The asterisk (*) denotes family members who have undergone genetic testing. II,2 – Mother, carrier of the *SH2D1A* variant. III,1 – Male, born in 2006, deceased at 11 months; postmortem diagnosis: HLH (affected sibling 1). III,2 – Female, 16 years old, carrier. III,3 – Male, 14 years old, healthy. III,4 – Male, born in 2012, deceased at 1 year 9 months; diagnosed with HLH (affected sibling 2). III,5 – Index case, male, born in 2023, deceased at 1 year; confirmed c.175del (p.Thr59Glnfs*22) variant in *SH2D1A* gene, hemizygous frameshift (affected sibling 3). III,6 – Male, born in 2025, healthy. **(B)** Structure of the *SH2D1A* gene. As a result of the deletion and frameshift, the amino acid sequence is completely different in wild type and mutated. The final protein is not only altered, but also extended by 14 amino acids. The asterisk (*) indicate stop codon. **(C)** Sequencing chromatogram showing the c.175del variant in the *SH2D1A* gene in a heterozygous state. **(D)** Sequencing chromatogram confirming the absence of the c.175del variant in the *SH2D1A* gene, leading to the p.Thr59Glnfs*22 frameshift mutation.

According to the family history, the first child in the family (affected sibling 1), born in 2006, died at the age of 11 months on the 9th day after hospitalization (**Figure 1A**). The disease had a rapid course, with fever, skin rashes, enterocolitis, pneumonia, hepatosplenomegaly, anemia, thrombocytopenia, and hypoproteinemia. Clinically, the diagnosis was fulminant hepatitis. The autopsy result showed signs of HLH.

The second child in the family, a girl, was born two years after the first child and is healthy. The third child, a boy, two years younger than the girl, is also alive and healthy. Both children have positive EBV serology without viremia.

The fourth child (affected sibling 2), a boy, was born two years later (in 2012). At 1 year and 9 months, he was hospitalized on the third day after disease onset with symptoms of fever, aphthous stomatitis, cheilitis, gingivitis, and widespread dermatitis (**Supplementary Table S1**). On the fifth day, he was transferred to the intensive care unit (ICU) due to progressing leukocytosis, hepatosplenomegaly, anemia, and hypertransaminasemia (**Supplementary Table S2**). On the tenth day of illness, tonic-clonic seizures were observed. The blood test revealed atypical mononuclears (17-51%). The IgM antibodies to cytomegalovirus (CMV) and EBV were detected, with negative IgG antibodies to EBV and without CMV viremia.

The results of the immunological examination of affected sibling 2 are presented in **Table 1**. A decrease in the relative number of CD3, CD4, and T-natural killers (CD3+CD56+), an increase in CD8, CD19, and a significant increase in the levels of IgA, IgM, as well as a decrease in the levels of complement C3 and C4 were observed.

**Table 1:** Immunological parameters in affected siblings 2 and 3.

Parameter	Affected sibling 2	Affected sibling 3	Normal range
Age	1 year, 9 months	1 year	
Day since the onset of clinical symptoms	10	13	
White blood cells, cells/µL	64300	14700	
Lymphocytes, cells/µL	39860	11030	
CD3, %	**23.5**	86.0	50-76
CD3, cells/µL	9367	9480	1800-6500
CD4, %	**3.9**	**18.0**	35-57
CD4, cells/µL	1554	2010	1200-4600
CD8, %	20.2	**68.0**	11-32
CD8, cells/µL	**8051**	**6450**	700-2400
CD4/CD8	**0.2**	**0.26**	0.95-2.25
CD19, %	**64.8**	**12.0**	17-32
CD19, cells/µL	**25829**	1320	500-2200
T-cell double negative (CD3+/CD4-/CD8-)		**0.80**	1.6-8.9
T-cell double positive (CD3+/CD4+/CD8+)		0.20	0.1-1.0
CD14	**1.8**		6-13
T-NK (CD3+CD56+), %	**0.2**	**2.6**	3-8
Natural killers (CD16/56), %	4.7	4.3	3-16
CD16/56 cells/µL	**1873**	470	100-900
IgA, g/l	**4.83**	**3.19**	0.20-1.00
IgM, g/l	**20.14**	**3.22**	0.19-1.46
IgG, g/l	7.18	5.13	4.53-9.16
IgE, IU/ml	<0.1		<60
Complement C3, g/l	**0.1**		0.9-1.8
Complement C4, g/l	**0.04**		0.1-0.4

Parameters that do not fall within the normal range are highlighted in bold.

Despite intensive treatment (oxygen therapy, mechanical ventilation, antibiotic therapy, acyclovir, glucocorticoid therapy at 10 mg/kg of prednisone, intravenous immunoglobulin (IVIG) 2g/kg, cytoplasm), anemia, hypertransaminasemia, hyperbilirubinemia, and coagulopathy progressed. The boy died on the 12th day from the onset of disease symptoms.

Reported here, affected sibling 3 continued to have a fever up to 39°C upon admission to the hospital. The child was in satisfactory nutritional status, weighing 9600 g. Pallor of the skin, tachycardia (heart rate 148 bpm), and hepatosplenomegaly were noted (spleen palpable +3 cm, liver +2 cm below the costal margin). Oxygen saturation was 95% on room air. Respiratory rate was 30 breaths per minute. No edema was present, and diuresis was adequate. No meningeal signs were detected.

**Figure 2:**
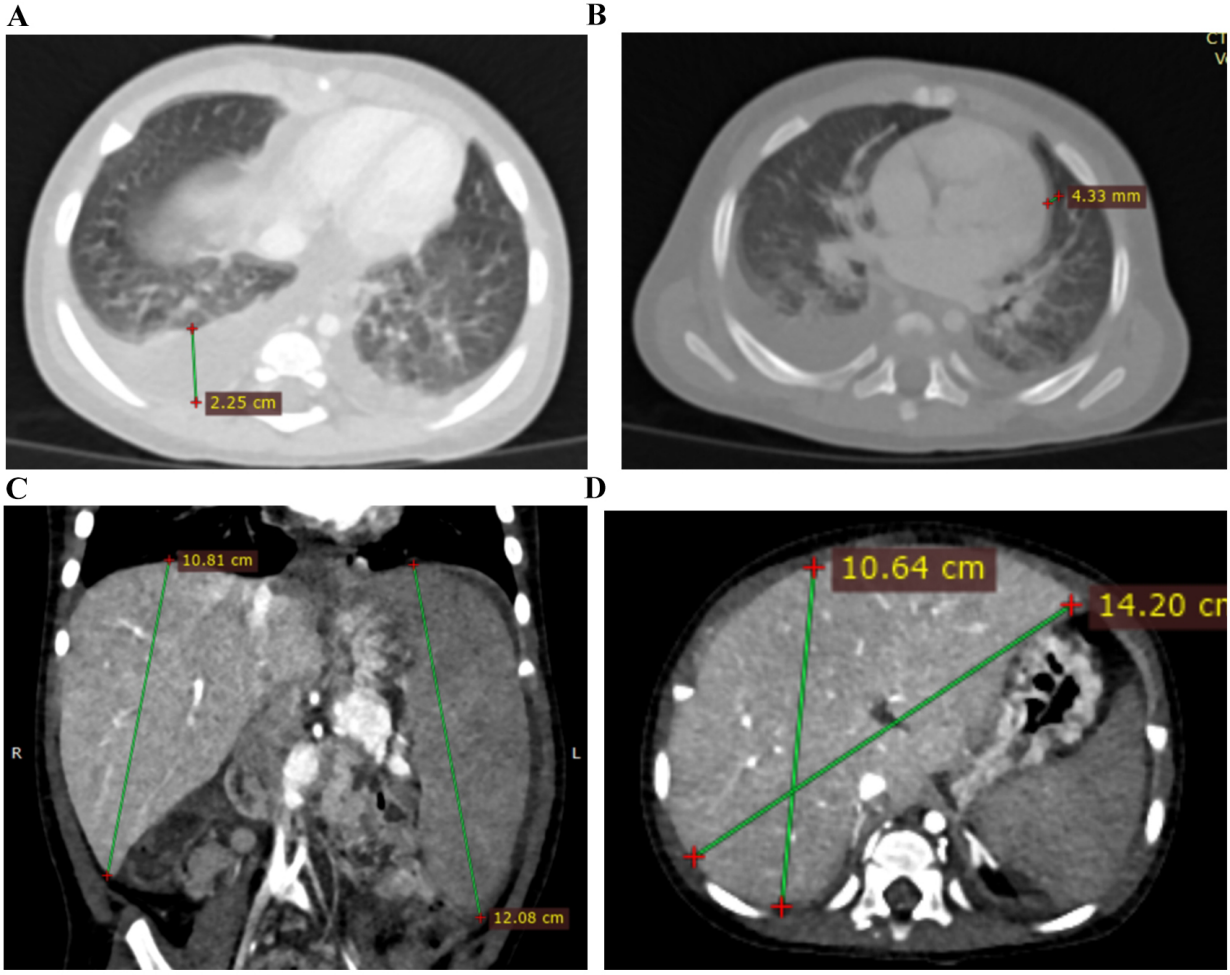
CT images of affected sibling 3 on 13 days from the disease onset. **(A)** Chest CT image showed bilateral ground-glass opacities and bilateral hydrothorax; **(B)** Chest CT image showed bilateral ground-glass opacities, bilateral hydrothorax, and pericardial effusion; **(C, D)** Abdominal CT images showed hepatosplenomegaly.

Laboratory findings revealed anemia, mild neutropenia, thrombocytopenia, elevated C-reactive protein (CRP), liver enzymes, lactate dehydrogenase (LDH), and decreased total protein and albumin levels (**Supplementary Table S3**, **Figure 2**). IgM to EBV was inconclusive, EBV-VCA-IgG was negative (0.31 U/mL), while IgM to CMV was positive. The rapid SARS-CoV-2 test was negative. Chest X-ray showed right-sided pneumonia. Immunoglobulin levels were IgA – 3.19 g/L, IgM – 3.22 g/L, IgG – 5.13 g/L.

Treatment included antibiotics (cefotaxime), glucocorticoids (dexamethasone 0.15 mg/kg), ganciclovir 5 mg/kg, IVIG 0.5 g/kg, and symptomatic therapy. Due to suspicion of primary immunodeficiency and lack of improvement, the child was transferred on day 3 to the pediatric intensive care unit of a tertiary care center.

The patient’s condition remained severe. Hepatosplenomegaly progressed (spleen +4 cm, liver +3 cm), anemia persisted (Hb – 71 g/L), alanine aminotransferase (ALT), aspartate aminotransferase (AST), and LDH remained elevated, and hypoproteinemia continued. Given the suspicion of an inborn error of immunity with immune dysregulation, blood was collected for genetic testing - primary immunodeficiency gene panel. Due to the risk of HLH, the dexamethasone dose was increased to 10 mg/m² (4 mg/day), IVIG 0.5 g/kg was repeated, and an erythrocyte transfusion was administered for worsening anemia. This led to partial stabilization: fever episodes decreased and did not exceed 38.0°C, neutrophil counts normalized, platelets showed mild fluctuations, hemoglobin levels stabilized, ferritin remained within normal range, and LDH levels decreased.

Considering a likely diagnosis of XLP1 and potential need for hematopoietic stem cell transplantation, the patient was transferred to a hospital with a transplantation unit on day 3 after admission to the tertiary hospital (day 8 of illness).

Upon admission, the child was drowsy, with pronounced pallor. Tachycardia and hepatosplenomegaly persisted (spleen +5–6 cm, liver +4 cm). Laboratory tests showed leukocytosis, severe anemia, high ESR (66 mm/h), and significantly elevated ALT and AST (**Supplementary Table S3**). Subsequently, respiratory distress developed, and thrombocytopenia progressed. Immunological evaluation (**Table 1**) revealed reduced CD4 and elevated CD8 cells, a decreased CD4/CD8 ratio, and decreased T-NK cells. IgA and IgM levels were elevated.

**Figure 3:**
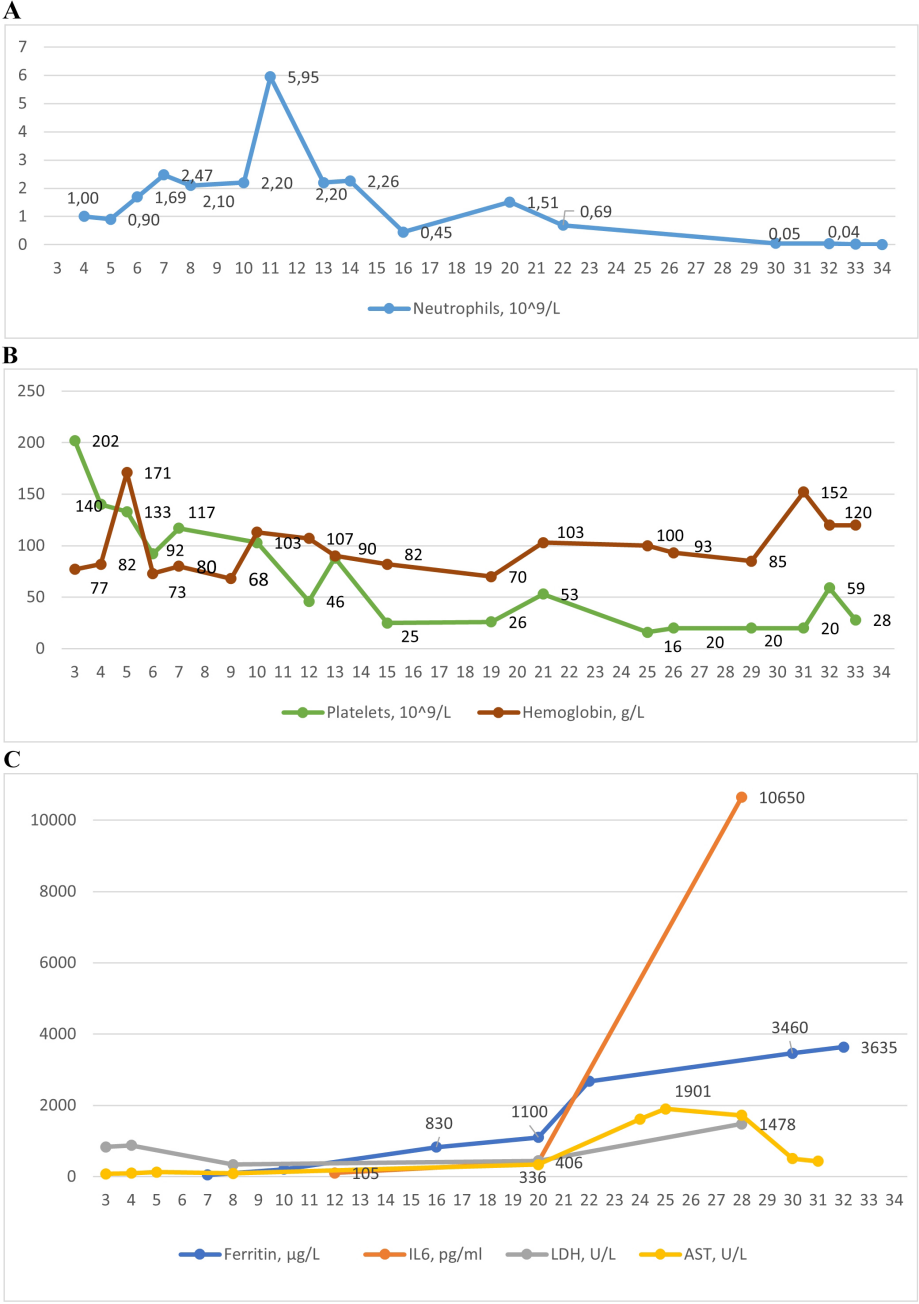
The dynamic of laboratory parameters in affected patient 3: **(A)** Absolute neutrophil count; **(B)** Platelets and hemoglobin counts; **(C)** Levels of ferritin, interleukin-6 (IL-6), lactate dehydrogenase (LDG), aspartate aminotransferase (AST).

CT scan showed bilateral ground-glass opacities, bilateral hydrothorax, moderate pericardial effusion, and hepatosplenomegaly (**Figure 3**).

Based on clinical, laboratory, and imaging findings, sepsis was suspected. Treatment was escalated to broad-spectrum antibiotics (imipenem/cilastatin and linezolid), glucocorticoids (methylprednisolone 3 mg/kg), ganciclovir, and symptomatic therapy.

Polymerase chain reaction (PCR) testing of blood revealed EBV DNA at 73,036 copies/10^5^ cells and HHV6 DNA at 24 copies/10^5^ cells. Anti–SARS–CoV–2 IgG antibodies were detected at 4,068 AU/mL and IgM at 577 AU/mL. A severe mixed viral infection was diagnosed. The ganciclovir dose was increased to 10 mg/kg, with continued supportive care.

Following ganciclovir therapy, EBV DNA decreased to 13,567/10^5^ cells; however, there was a sharp rise in neutropenia, thrombocytopenia, and anemia, along with hyperferritinemia, increased ALT, a particularly marked increase in AST, elevated LDH, bilirubin, triglycerides, and interleukin-6 (IL-6) (**Supplementary Table S3**, **Figure 2**), suggesting the development of HLH.

Bone marrow examination revealed reduced granulocytic lineage, preserved erythroid lineage with dysplastic changes (61% megaloblasts), expansion of the megakaryocytic lineage with dysplasia, and elevated lymphocytes without signs of hemophagocytosis. Genetic test results were still pending at the time.

Differential diagnosis included sepsis, severe EBV infection with SARS-CoV-2, and secondary HLH in the context of immunodeficiency. The child continued receiving broad-spectrum antibiotics, corticosteroids (methylprednisolone 3 mg/kg), and replacement therapy with platelet concentrates, erythrocyte transfusions, cryopreserved plasma, albumin, and symptomatic treatment. Immunosuppressive therapy with etoposide and cyclosporine was not initiated due to the strong clinical suspicion of sepsis.

On day 30 of illness, the patient’s condition deteriorated rapidly. Seizures, respiratory arrest, and coma developed. Respiratory rate was 45–50 breaths/min, SpO_2_ was 80–90% on 4 L/min oxygen. The child was transferred to mechanical ventilation. Severe pancytopenia and multiple organ failure developed.

Tragically, the intensive care unit of the hospital, where the child was treated, was severely damaged during a massive Russian missile attack. The patient was urgently transferred to another clinic, where he died in the following days.

The results of genetic testing were received on the second day after the child’s death. A frameshift variant c.175del (p.Thr59Glnfs*22) in *SH2D1A* was identified, hemizygous, classified as likely pathogenic, and associated with XLP1 (**Figures 1B–D**). To our knowledge, this variant has not been reported in the clinical literature in affected individuals. Further family screening revealed that the mother and sister are carriers of this variant, while two siblings (boys) are healthy (**Figure 1A**).

## Discussion

We present a clinical case of fatal HLH due to a novel frameshift variant c.175del (p.Thr59Glnfs*22)* in *SH2D1A*, hemizygous, classified as likely pathogenic, associated with XLP1. The c.175del deletion in the SH2D1A gene leads to the removal of one nucleotide at position 175, causing a frameshift. This results in the formation of a truncated protein with an abnormal amino acid sequence, known as p.Thr59Glnfs22. Such a protein is either non-functional or unstable, negatively affecting its role in immune system regulation. To our knowledge, this variant has not been reported in the clinical literature in affected individuals. The genetic diagnosis was confirmed only in the third affected patient from this family. Although in the two previous deceased siblings, it was not possible to confirm the genetic diagnosis, the rapid progression of the disease leading to HLH suggests a likely XLP1.

The manifestation of symptoms in our case was early, in one year. In another multicenter study, the median age of presentation was 4 years (11). Upon admission to the clinic, the patient had hepatosplenomegaly, anemia, thrombocytopenia, and hypoproteinemia. Certain differences in clinical symptoms may be observed even in one family (11, 13).

Data from other studies did not reveal genotype-phenotype correlations (11, 14). Moreover, there is a report of a new variant in the *SH2D1A* gene (c.49G > A (p.E17K)), which was found in a 21-year-old patient with fatal EBV infection-associated HLH (13). This variant showed normal expression of the SAP protein, although binding to the phosphorylated CD244 receptor was reduced by >95%. Additionally, three healthy brothers, two of whom were seropositive for EBV, carried the same variant in the *SH2D1A* gene, further demonstrating the influence of gene expression on the clinical course of the disease, which accounts for the varied clinical presentation within a family.

It should be noted that in the first affected sibling, studies to determine EBV viremia were not conducted. Clinically, hepatitis was the primary manifestation, and lymphohistiocytosis was confirmed postmortem histologically. In the second affected sibling, serological tests confirmed active CMV and EBV, although PCR was only performed for CMV, yielding a negative result. The absence of IgG to EBV indicated primary infection. In the third affected sibling, high EBV viremia and positive serological tests (IgM and IgG) for SARS-CoV-2 were confirmed. Thus, while the issue of co-infection in the second affected sibling might be uncertain, the combination of COVID-19 is evident in the third affected sibling.

In the early studies on XLP1, only the role of EBV in disease development was clearly recognized (15–17), but more recent research also points to the potential role of other pathogens (11, 18). Specifically, Chung et al. described a 5-year-old Nepalese boy with XLP1, presenting with agammaglobulinemia and SARS-CoV-2 infection, who died of diffuse alveolar damage 22 days after admission during the SARS outbreak (19). The role of SARS-CoV-2 in the development of HLH is also noted in other immunodeficiencies (20, 21).

Susceptibility to EBV in patients with XLP is associated with EBV infection of B-cells and the inability of SAP-deficient CD8+ T-cells to respond (2). It was hypothesized that patients with XLP would be susceptible to viruses that use B-cells as hosts, but data on such viruses are limited.

However, only 64.6% of patients with XLP1 due to *SH2D1A* variants were EBV-positive at presentation or diagnosis (11). While EBV was indeed the cause of the severe disease course, most EBV-positive patients (77.8%) died within 2 months of presentation due to disease progression (11). Additionally, EBV was responsible for a higher incidence of HLH manifestation (51% vs. 21% in EBV-negative patients). Mortality was also lower in EBV-negative patients (28.6%). Studies have shown that EBV-HLH typically leads to impaired CD8+ T-cell proliferation and enhanced type I IFN signaling, regardless of patient origin, highlighting key features of EBV-HLH (22). XLP1 is now considered more of a disorder of immune dysregulation, not only triggered by EBV.

The immunologic parameters in the reported case demonstrated a low percentage of CD4, high CD8 levels, a low CD4/CD8 ratio, normal CD19 levels, and slightly reduced T-NK. Similar changes were reported in other affected children with XLP1 (19, 23).

Another multicenter study showed that among 47 patients, 19 showed a reduced percentage of B cells, 26 had low NK cell numbers, and 12 had a reversed CD4:CD8 ratio (11). Some studies suggest that 10% of affected boys exhibit immunological abnormalities before any signs of EBV impact are observed (24).

Studies show that in the development of HLH, there is usually a significant increase in CD8+ T-cells (24), which was also observed in our study. A decrease in NK cell numbers does not always reflect their function, so functional studies are required. SAP is necessary for the development of normal invariant NKT cells (iNKT) and for normal cell death induced by T-cell restimulation (RICD) (25, 26). The ability to assess these cells and conduct RICD can be used to confirm NK cell dysfunction.

Direct screening for SAP shows 87% sensitivity and 89% specificity for predicting pathological mutations in *SH2D1A* (25).

Regarding immunoglobulins, we observed increased levels of IgA and IgM. Other studies have shown agammaglobulinemia or hypogammaglobulinemia in patients with XLP1 (19). However, another study reported similar results in a child with XLP1 and the development of HLH (23). The difference may be due to the phase of the disease at the time of the study.

Another important issue is the diagnosis of HLH. Clinical and laboratory findings in affected siblings 2 and 3 met the HLH-2004 diagnostic criteria (27), including persistent high fever, splenomegaly, cytopenia involving all three blood cell lineages, hypertriglyceridemia, hypofibrinogenemia, and hyperferritinemia. Additional findings included elevated AST, LDH, and neurological manifestations such as seizures and coma.

However, the patient’s critical condition, marked leukocytosis, pneumonia, and hepatosplenomegaly initially led clinicians to suspect sepsis before the appearance of clear HLH signs. As a result, immunosuppressive therapy with etoposide and cyclosporine was not initiated. Thus, our case also highlights the need to improve physician awareness regarding the diagnosis and management of HLH (28).

Although mortality from EBV-associated HLH in patients with XLP1 has decreased in recent years, it remains relatively high, at 65.6%, according to a multicenter study (11). The median age at presentation was 3 years and 2 months (range 8 months to 9 years).

Bone marrow transplantation remains the only curative treatment for XLP1, even in the development of HLH. Specifically, bone marrow transplantation in patients with HLH was successful in 50% of cases, compared to an 18.8% survival rate for those who were not transplanted after HLH (11). Therefore, early diagnosis of XLP1 before EBV exposure may help improve outcomes for patients (28).

The patient’s family experienced the devastating loss of three sons due to the same severe illness, which remained undiagnosed until the most recent case. Only through genetic analysis did they finally understand the underlying cause — a novel pathogenic variant in *SH2D1A*. This finding had valuable insight for future family planning.

Following the death of the index patient, the family had another son. Thanks to the established diagnosis, genetic testing was performed twice — prenatally and at birth — confirming that the newborn was not affected. Additionally, the daughter, identified as a carrier of the variant, was evaluated by both a clinical geneticist and an immunologist, and appropriate recommendations were provided for future planning.

The family consented to the publication of this case to raise awareness of primary immunodeficiencies and to emphasize the importance of early genetic diagnosis and counseling, particularly in families with a history of immune dysregulation or early childhood deaths.

The limitations of this study include the retrospective analysis of data in the affected siblings, the lack of laboratory examinations in the first affected sibling, and certain examinations in the second sibling (EBV-DNA) that did not allow for full determination of the cause of HLH. Another limitation is the inability to conduct extended functional tests that could have facilitated earlier diagnosis of the disease. Furthermore, the result of genetic testing was received postmortem, precluding the timely initiation of targeted therapy.

## Conclusions

This study presents a novel *SH2D1A* frameshift variant associated with XLP1 and manifesting as fatal HLH in early age. The clinical presentation of affected siblings showed variability, underscoring the complexity of disease expression even within the same family. Our findings support previous reports that EBV is a key trigger for HLH in XLP1, but co-infections with other pathogens such as SARS-CoV-2 may also play a role. Despite advances in HLH treatment, mortality remains high, emphasizing the importance of early genetic diagnosis before EBV exposure. Genetic diagnosis revealed the root cause, and carrier testing will bring future cases to medical intervention faster. Bone marrow transplantation remains the only curative approach for XLP1, with improved outcomes in those diagnosed and treated early.

## Data Availability

The datasets presented in this study can be found in online repositories. The names of the repository/repositories and accession number(s) can be found in the article/**Supplementary Material**.
