# Supplementary material for: Fatal HLH in patients with X-linked lymphoproliferative disease 1 due to a novel variant in SH2D1A: case report
Source: Front Immunol. 2025 May 19;16:1602107. doi: 10.3389/fimmu.2025.1602107 (PMC12127284; doi:10.3389/fimmu.2025.1602107)
Supplement: Supplementary file 1 [file Table1.docx]

**Table S1.** Clinical presentation of affected siblings

| **Parameter** | Affected sibling 1 | Affected sibling 2 | Affected sibling 3 |
| --- | --- | --- | --- |
| Fever | Yes | Yes | Yes |
| Rash | Yes | Yes | No |
| Aphthous stomatitis | No | Yes | No |
| Gingivitis | No | Yes | Yes |
| Enterocolitis | Yes | Yes | No |
| Anemia | Yes | Yes | Yes |
| Thrombocytopenia | Yes | Yes | Yes |
| Hypoproteinemia | Yes | Yes | Mild |
| Hepatosplenomegaly | Yes | Yes | Yes |
| Lymphoproliferative disorder | Yes | Yes | No |
| Pneumonia | Yes | Yes | Yes |
| Meningoencephalitis | No | Yes | Yes |
