# Supplementary material for: Fatal HLH in patients with X-linked lymphoproliferative disease 1 due to a novel variant in SH2D1A: case report
Source: Front Immunol. 2025 May 19;16:1602107. doi: 10.3389/fimmu.2025.1602107 (PMC12127284; doi:10.3389/fimmu.2025.1602107)
Supplement: Supplementary file 2 [file Table2.docx]

**Table S2.** Laboratory test results of the affected sibling 2

| Indicator / Day from onset of symptoms | 4 | 7 | 8 | 10 | 11 | 12 | Reference range |
| --- | --- | --- | --- | --- | --- | --- | --- |
| EBV IgM |  |  |  | pos |  |  |  |
| CMV IgM |  |  |  | pos |  |  |  |
| WBC, 10^9^/L | **25.7** | **41.6** | **59.4** | **64.3** | **60.8** | **51.4** | 4.0-10.0 |
| Neutrophils, 10^9^/L | 5.39 | 12.06 | 14.85 | 5.14 | 15.2 | 4.62 | 1.5-7.0 |
| Lymphocytes, 10^9^/L | 3.34 | 17.05 | 22.57 | 39.87 | 43.16 | 37.01 | 2.0-6.5 |
| Monocytes, % | 11 | 11 | 5 | 11 | 4 | 18 | 0-10 |
| Atypical mononuclear cells, % | 51 | 17 | 32 |  |  |  | 0 |
| Platelets, 10^9^/L |  |  | **139** |  |  | 169 | 150-400 |
| Hemoglobin, g/L | **110** | **109** | **103** | **89** | **71** | **61** | 120-140 |
| ESR, mm/h | 5 | **17** | **16** | **17** | **43** | **32** | 0-10 |
| CRP, mg/L |  |  |  |  | 35.6 |  | <5 |
| AST, U/L |  |  | **93** |  | **2554** |  | <40 |
| ALT, U/L |  |  | **73** |  | **602** |  | <37 |
| GGT, U/L |  |  |  |  | **336** |  | <55 |
| Bilirubin, μmol/L |  |  | **62.2** |  | **68.2** |  | <21 |
| Albumin, g/L |  |  |  |  | **17.8** |  | 35-52 |
| Total protein, g/L |  |  | **47.7** |  | **51.7** |  | 60-83 |
| Creatinine, μmol/L |  |  | 38 |  | 18 |  | 70-100 |
| Fibrinogen, g/L |  |  |  | **0.15** | **ND** |  | 2.0-4.0 |
| PT, sec |  |  |  | **18** | **29.1** |  | 13-17 |
| INR |  |  |  | **2.86** | **3.67** |  | 0.8-1.2 |
| Prothrombin, % |  |  |  | **33** | **22** |  | 70-100 |

EBV - Epstein-Barr virus; PCR – polymerase chain reaction; CMV – cytomegalovirus; WBC – white blood count; CRP – C-reactive protein; IL6 - interleukin-6; AST - aspartate aminotransferase; ALT - alanine aminotransferase; GGT - gamma-glutamyl transferase; LDH- lactate dehydrogenase; TGL – triglycerides; PT – prothrombin time; INR – international normalized ratio. Indicators that do not fall within the normal range are highlighted in bold.
