# Supplementary material for: Fatal HLH in patients with X-linked lymphoproliferative disease 1 due to a novel variant in SH2D1A: case report
Source: Front Immunol. 2025 May 19;16:1602107. doi: 10.3389/fimmu.2025.1602107 (PMC12127284; doi:10.3389/fimmu.2025.1602107)
Supplement: Supplementary file 3 [file Table3.docx]

**Table S3.** Investigations and treatment in the affected sibling 3

| Indicator / Day from onset of symptoms | 4 | 5 | 6 | 7 | 8 | 10 | 11 | 13 | 14 | 16 | 20 | 22 | 26 | 27 | 30 | 32 | 33 | 34 | Reference range |
| --- | --- | --- | --- | --- | --- | --- | --- | --- | --- | --- | --- | --- | --- | --- | --- | --- | --- | --- | --- |
| EBV IgM |  | dbt |  |  |  |  |  |  |  |  |  |  |  |  |  |  |  |  |  |
| PCR, EBV |  |  |  |  |  |  |  | **pos** |  |  | **pos** |  |  |  |  |  |  |  |  |
| CMV IgM |  | pos |  |  |  |  |  |  |  |  |  |  |  |  |  |  |  |  |  |
| SARS-CoV-2, IgM |  |  |  |  |  |  |  |  | **pos** |  |  |  |  |  |  |  |  |  |  |
| WBC, 10^9^/L | 4.8 | 5.6 | 4.03 | 5.85 | 10.2 | **18.3** | **23.8** | **14.7** | 7.8 | 9 | 6.6 | 4.9 | **2.2** | **3.6** | **1.2** | **1.76** | **0.52** | **0.65** | 4.0-10.0 |
| Neutrophils, % | 20.0 | **16.3** | 42 | 42 | 21 | **12** | 25 | **14** | 29 | **5** | 23 | **14** |  |  | **4** | **2** | **4** | **2** | 20-40 |
| Neutrophils, 10^9^/L | **1.0** | **0.9** | 1.69 | 2.47 | 2.1 | 2.2 | 5.95 | 2.2 | 2.26 | **0.45** | 1.51 | **0.69** |  |  | 0.05 | 0.04 | 0.02 | 0.01 | 1.5-7.0 |
| Lymphocytes, 10^9^/L | 3.7 | 3.4 | 1.76 | 1.89 | 6.68 | 13.9 | 15.5 | 11.02 | 3.43 | 7.1 | 4.48 | 3.67 |  |  | 1.1 | 1.59 | 0.41 | 0.55 | 2.0-6.5 |
| Monocytes, % |  | 3 | 6 |  |  | 10 | 9 | 7 |  | **15** | 6 | 10 |  |  | 7 | 5 | 13 | 5 | 1-10 |
| Platelets, 10^9^/L | 202 | **140** | **133** | **92** | **117** |  | **103** | **46** | **88** | **25** | **26** | **53** | **16** | **20** | **20** | **20** | **59** | **28** | 150-400 |
| Hemoglobin, g/L | **77** | **82** | **71** | **73** | **80** | **68** | **113** | **107** | **90** | **82** | **70** | **103** | **100** | **93** | **85** | 152 | 120 | 120 | 120-140 |
| ESR, mm/h |  | **19** | 8 | **17** |  | **66** | **27** |  |  | **63** | **49** | **37** |  |  | **14** |  |  |  | 0-10 |
| CRP, mg/L |  | **33** | **6.5** | **12.4** |  | **22** |  |  |  |  |  | **36** |  |  | **91** | **64** | **46** |  | <5 |
| Ferritin, μg/L |  | 18.2 |  | 45 |  | 209 |  |  |  | **830** | **1100** | **2677** |  |  | **3460** | **3635** |  |  | 24-336 |
| IL6, pg/ml |  |  |  |  |  |  |  |  | **104.5** |  |  | **406** |  |  | **10650** |  |  |  | <7 |
| AST, U/L |  | **77** | **95** | **127** |  | **93** |  |  |  |  |  | **336** | **1612** | **1901** | **1719** | **503** | **429** |  | <40 |
| ALT, U/L |  | **71** | **61** | **74** |  | **62** |  |  |  |  |  | **95** | **314** | **348** | **381** | **178** | **109** |  | <37 |
| GGT, U/L |  |  | 22 |  |  | **217** |  |  |  |  |  | **2568** |  |  | **2060** |  |  |  | <55 |
| LDH, U/L |  | **833** | **875** |  |  | **339** |  |  |  |  |  | **443** |  |  | **1478** |  |  |  | 140-280 |
| Bilirubin, μmol/L |  | 4.5 | 4.8 |  |  | 3.7 |  |  |  |  |  | **74.8** | **134** | **147** | **213** | **260** | **256** |  | <21 |
| Albumin, g/L |  | **33.2** | **26.4** |  |  |  |  |  |  |  |  |  |  |  |  |  |  |  | 35-52 |
| Total protein, g/L |  | **50** | **52** |  |  | **56** |  |  |  |  |  | 65 | **57** | 64 |  |  |  |  | 60-83 |
| TGL, mmol/l |  | 1.5 |  |  |  | **2.95** |  |  |  | **3.98** | **5.63** |  |  |  |  |  |  |  | <1.7 |
| Creatinine, μmol/L |  | 38 | 28 |  |  | 34 |  |  |  |  |  | 22 | 27 |  |  | 12 |  |  | 70-100 |
| Fibrinogen, g/L |  | **1.99** | 2.43 |  |  |  |  |  |  |  |  |  |  |  |  |  |  |  | 2.0-4.0 |
| PT, sec |  | 14.4 | 12.2 |  |  |  |  |  |  |  |  |  |  |  |  |  |  |  | 13-17 |
| Prothrombin, % |  | 87.9 | 98.4 |  |  |  |  |  |  |  |  |  |  |  |  |  |  |  | 70-100 |
| **Other investigations** |  | X-ray |  |  |  |  |  | CT |  |  | BMB |  |  |  |  |  |  |  |  |
| **Treatment*** |  |  |  |  |  |  |  |  |  |  |  |  |  |  |  |  |  |  |  |
| Antibiotic therapy | Сefotaxime | | | | | | Imipenem/cilastatin + linezolid | | | | | | | | | Сolistin + meropenem | | |  |
| Glucocorticoids | Dex 0.15 mg/kg  (1.5 mg/day) | | | Dex 10 mg/m²  (4 mg/day) | | Methylprednisolone 30 mg/day | | | | | | | | | | | | |  |
| IVIG, g/kg |  | 0.5 |  | 0.5 |  | 0.5 |  |  |  |  |  |  |  |  |  |  |  |  |  |
| Ganciclovir |  | 5 mg/kg | | | | | 10 mg/kg | | | | | | | | | | | |  |

EBV - Epstein-Barr virus; PCR – polymerase chain reaction; CMV – cytomegalovirus; WBC – white blood count; CRP – C-reactive protein; IL6 - interleukin-6; AST - aspartate aminotransferase; ALT - alanine aminotransferase; GGT - gamma-glutamyl transferase; LDH- lactate dehydrogenase; TGL – triglycerides; PT – prothrombin time; CT – computer tomography; BMB – bone marrow biopsy; Dex – dexamethasone. Indicators that do not fall within the normal range are highlighted in bold, * The table summarizes the main treatment the patient received and does not include replacement therapy with platelet concentrates, erythrocyte transfusions, cryopreserved plasma, albumin, and symptomatic treatment.
